# The Rural Workhouse Infirmary

**Published:** 1914-05-16

**Authors:** 


### THE RURAL WORKHOUSE INFIRMARY.

The publicity given by the Report of the Royal Commission on the Poor Laws to the statements made by numerous witnesses as to the deplorable conditions existing in many rural workhouse infirmaries has led in the intervening years to the introduction of many improvements. That much still remains to be done in a large number of instances is shown, however, by a recent report on the Chelmsford Workhouse Infirmary by Captain Hervey, one of the Local Government Board's inspectors. In the course of his report the inspector says: "The infirmary was built in 1849, and, as was usual in those days, there were practically no sanitary arrangements; alterations had been subsequently made, which were still very limited and inadequate. In 1868 an addition was made, but the accommodation for the sick was the oldest and worst accommodation in this workhouse; it was far below the general standard, and altogether unworthy of a Union of the importance of Chelmsford. It was entirely lacking in all modern hospital convenience, and did not lend itself to economical or convenient nursing of sick or infirm persons. In the best interests of the Union and Poor Law generally, the Guardians would be well advised to abandon the building eventually as an infirmary, and to devote the contemplated expenditure to the nucleus of a modern infirmary. There seemed to be every justification for proceeding with the building of a new infirmary without any delay."

This report was discussed at the last meeting of the Board of Guardians. It is astonishing to find that the existing state of affairs found several defenders, but after some discussion the report was referred to the house committee for consideration. The problem of these small workhouses is a very serious matter, and one that calls urgently for reform. With the present small Poor-Law areas an efficient solution, except at a cost the ratepayers can hardly be expected to assent to, is practically impossible. It is only by an amalgamation of Unions and parishes so as to provide one large and properly equipped infirmary for the whole area that the sick poor can receive the medical treatment on modern hospital lines which mere humanity demands. And, as we have said over and over again, the humane and efficient treatment of the sick is, in the long run, the only course consistent with economy. It is cheaper to restore the sick to health than to maintain them as chronic invalids, or to allow them to die and have to provide for their dependants.

